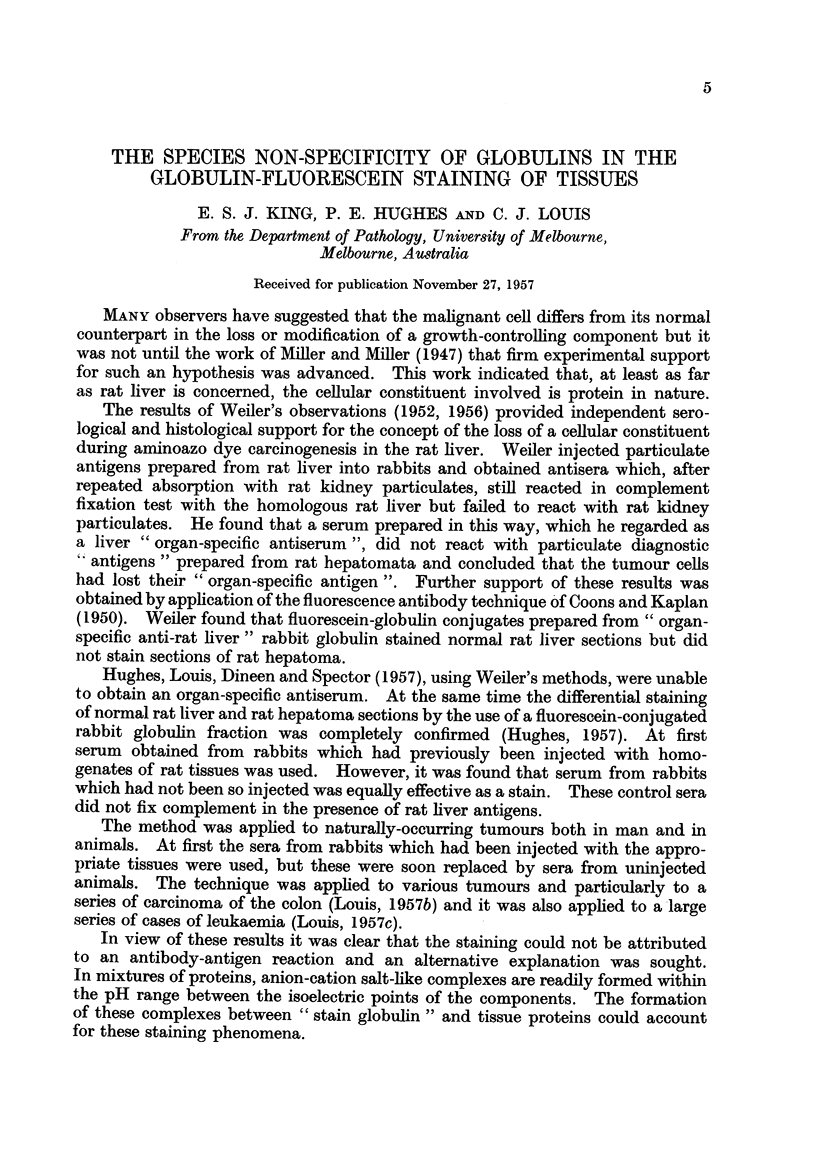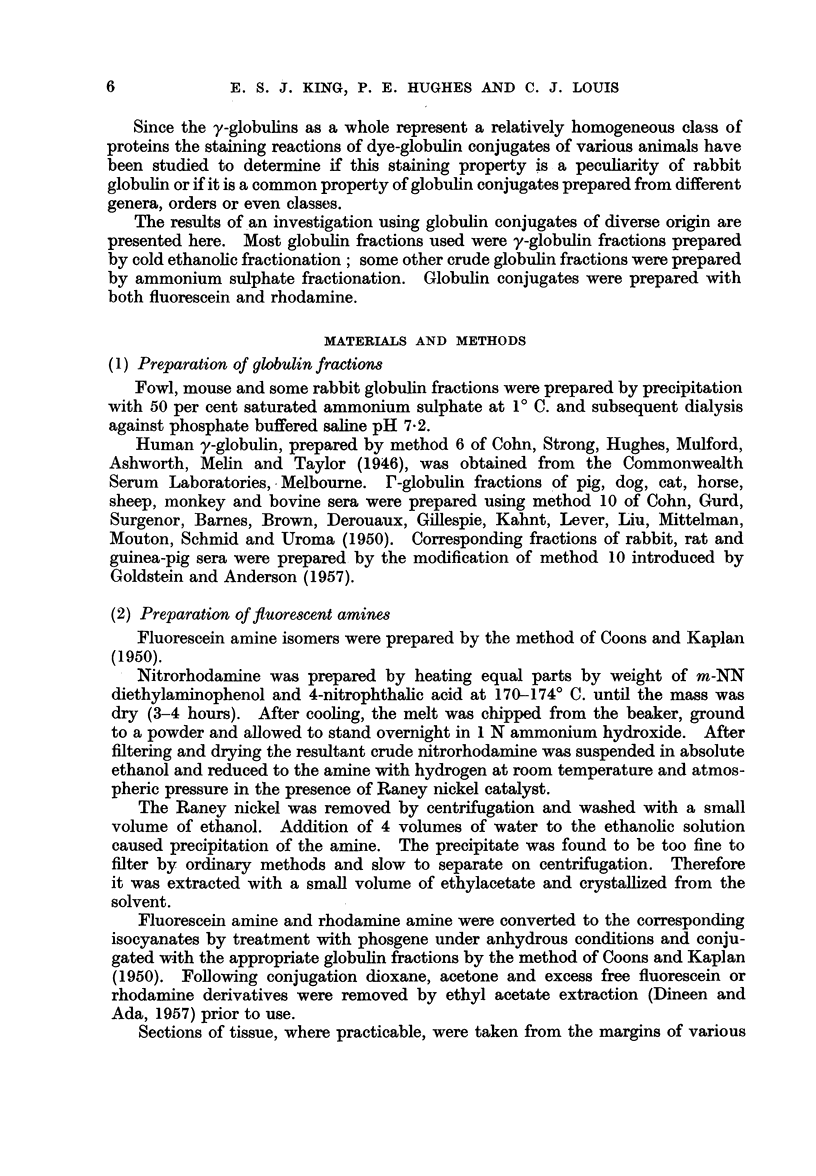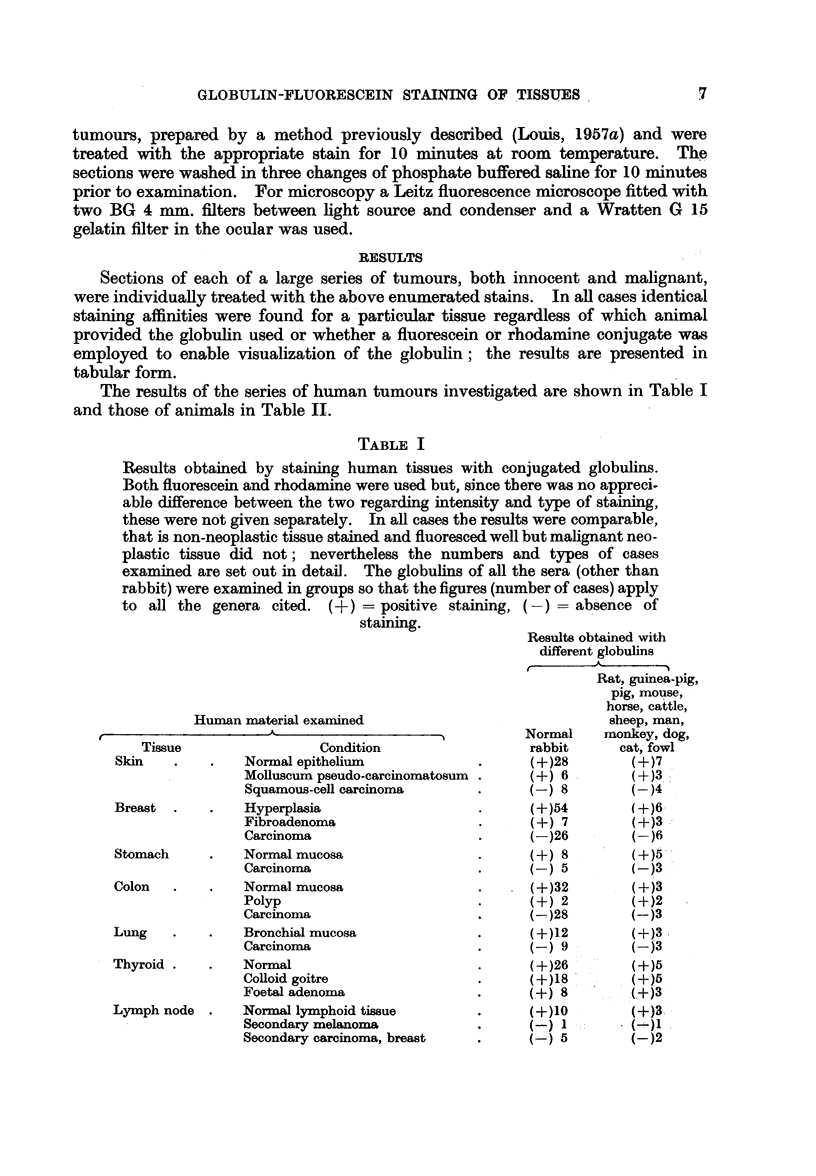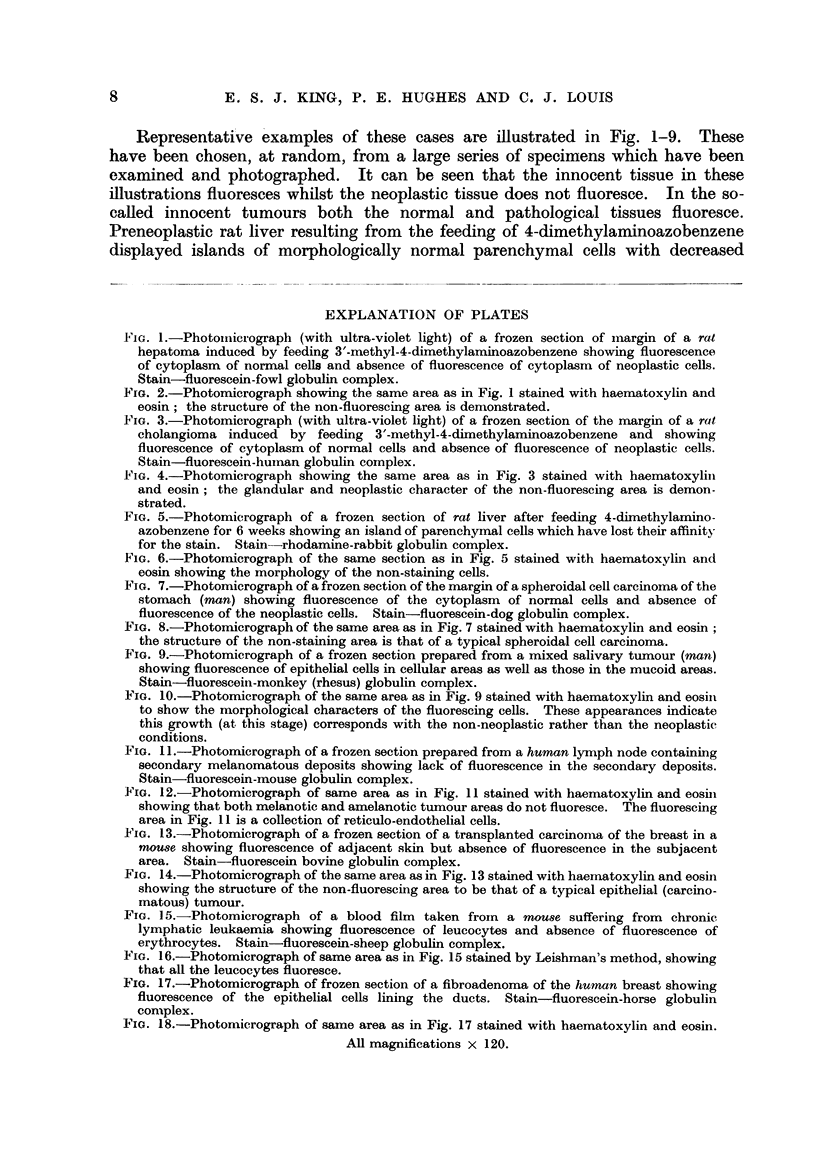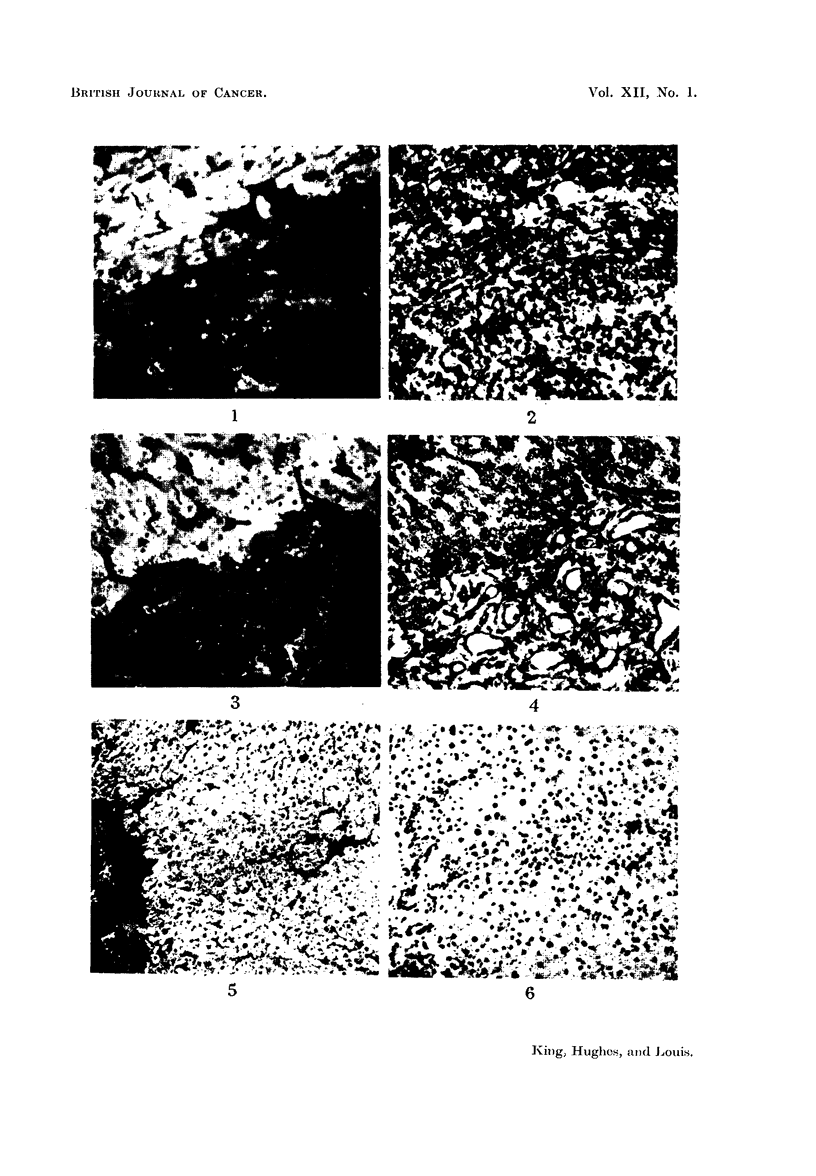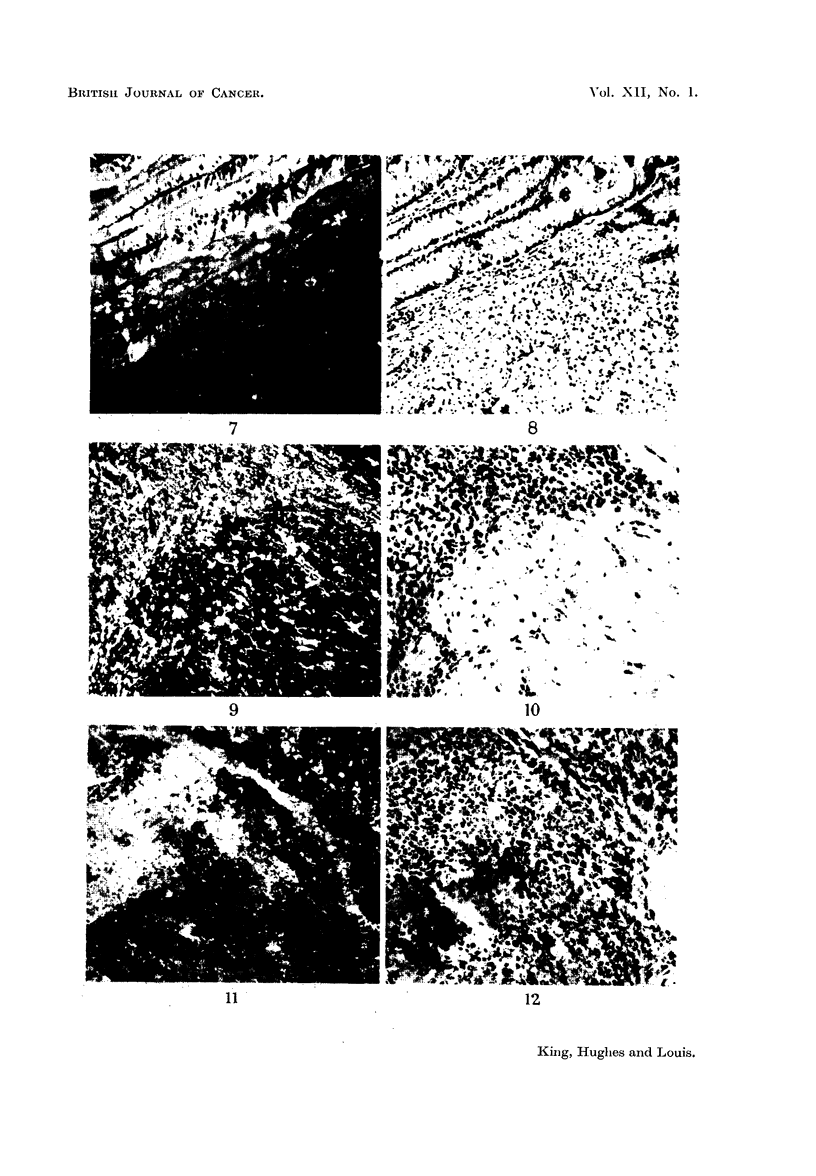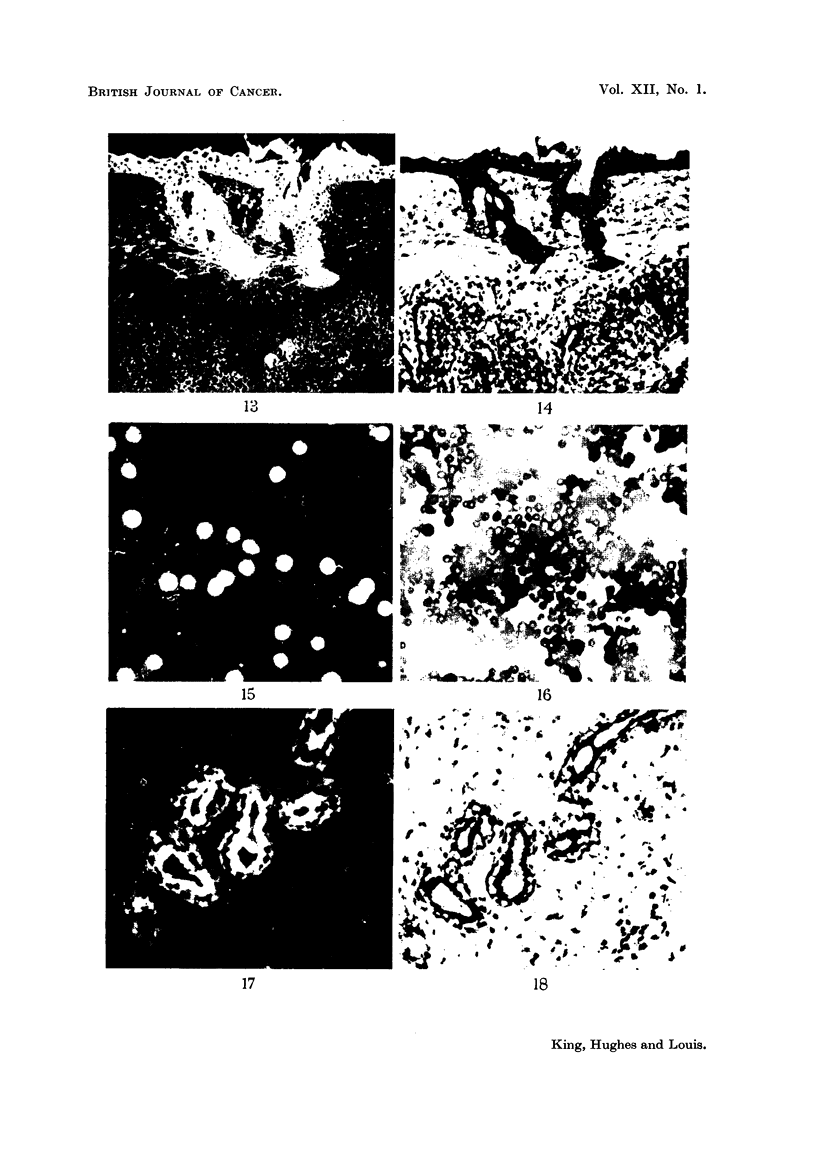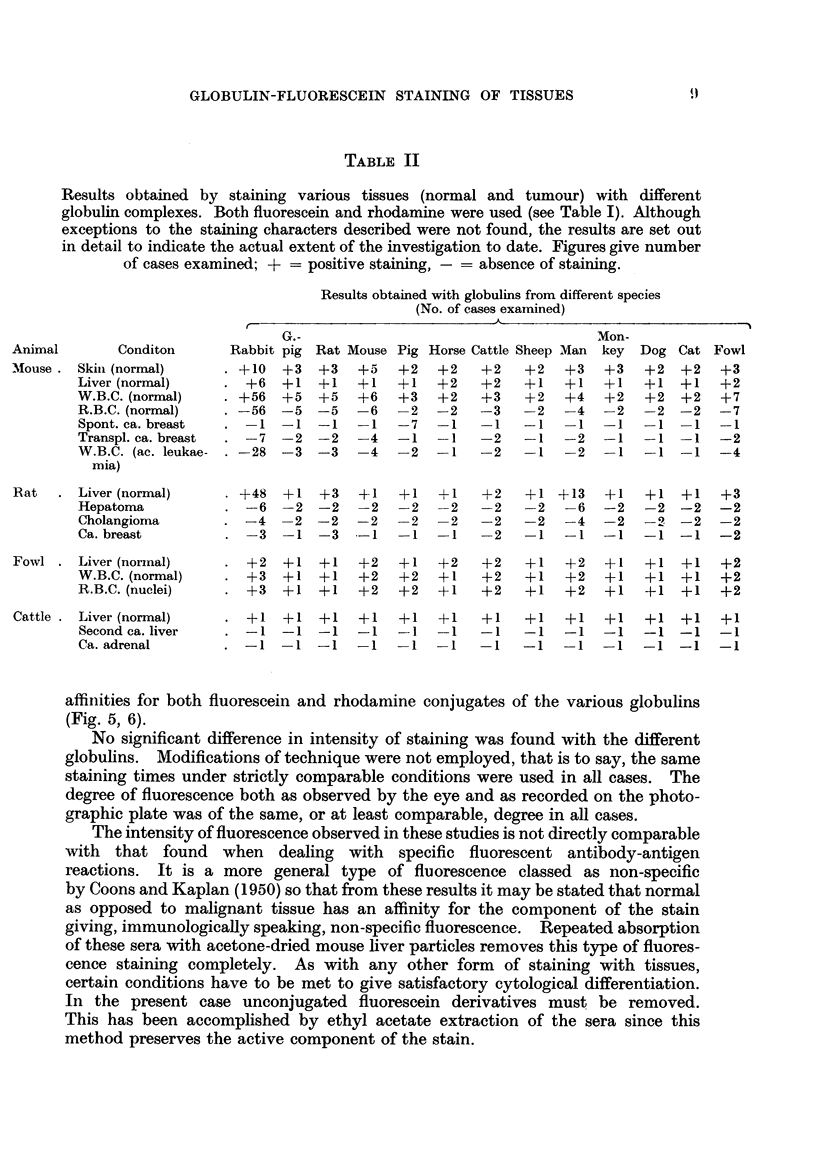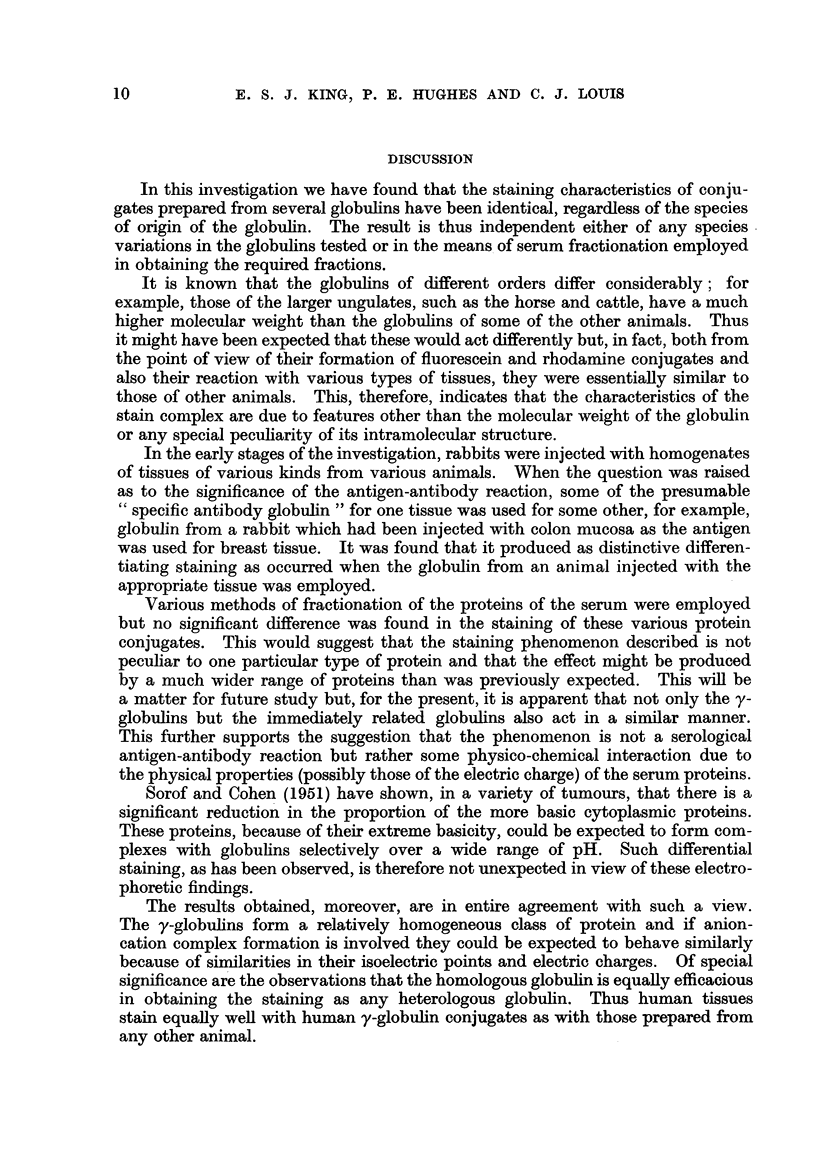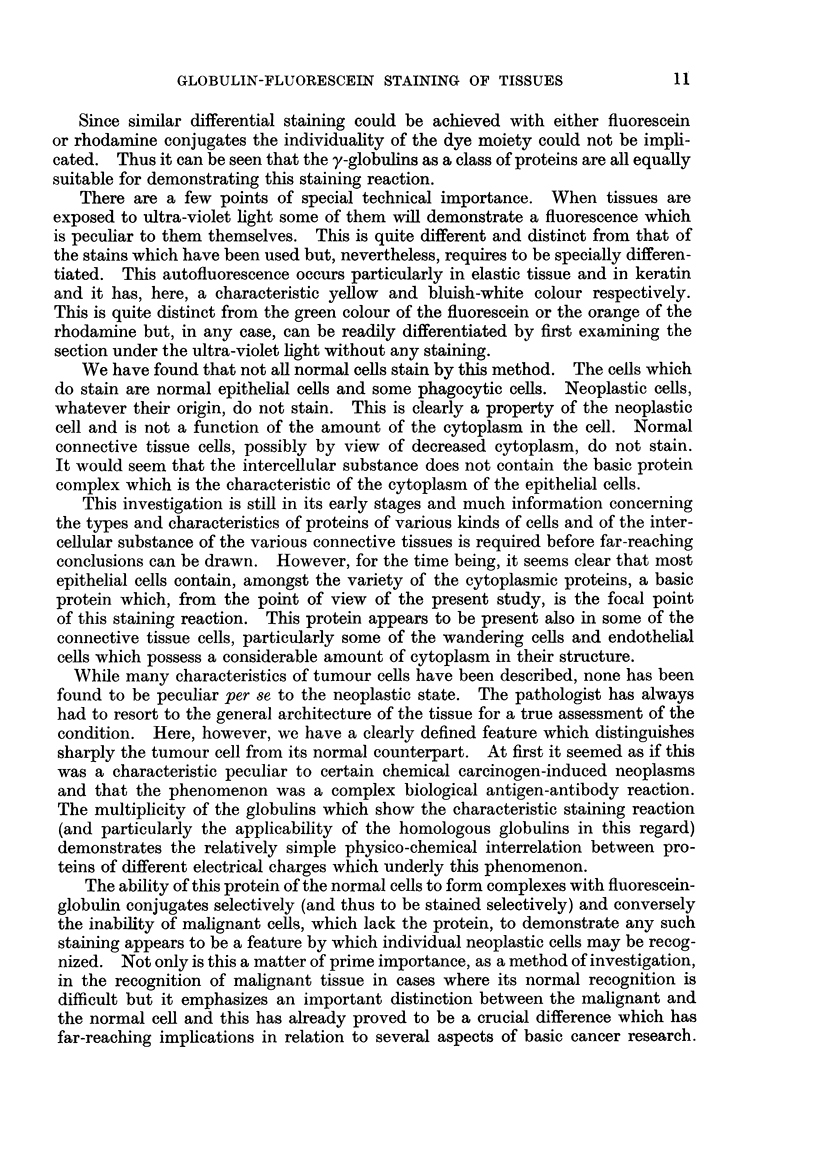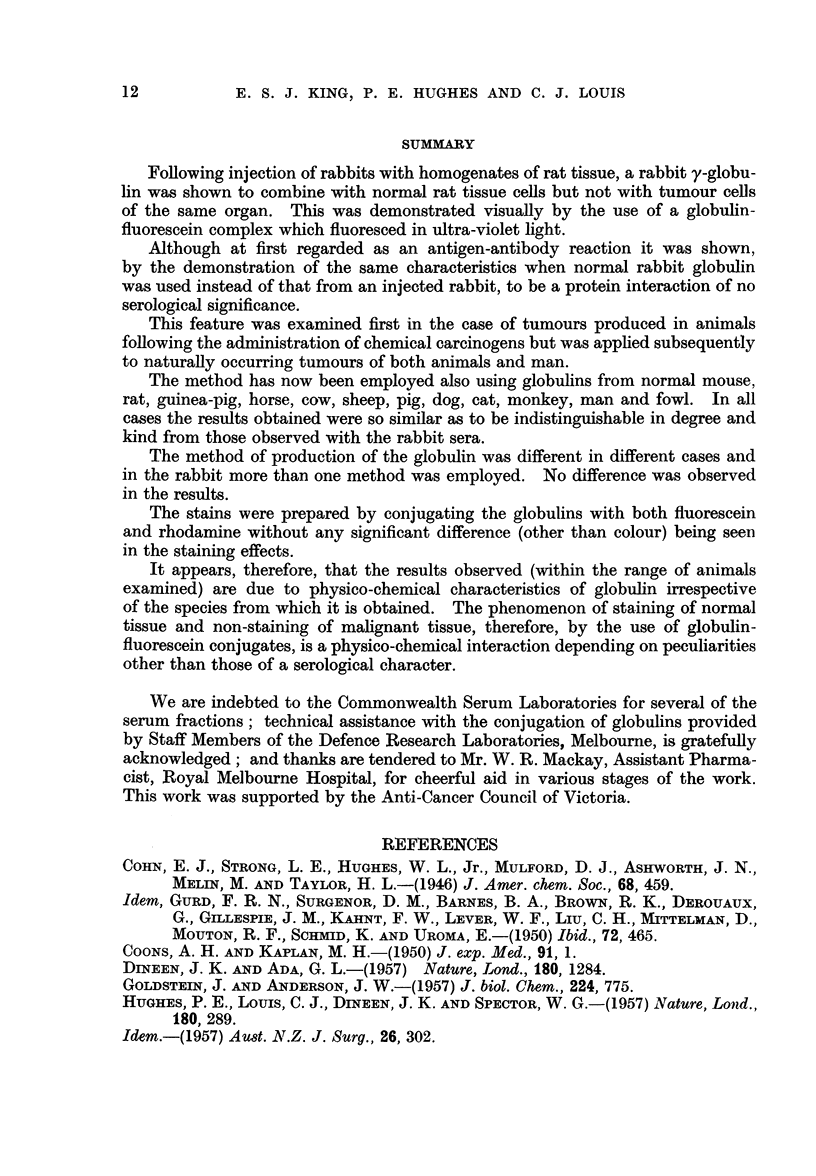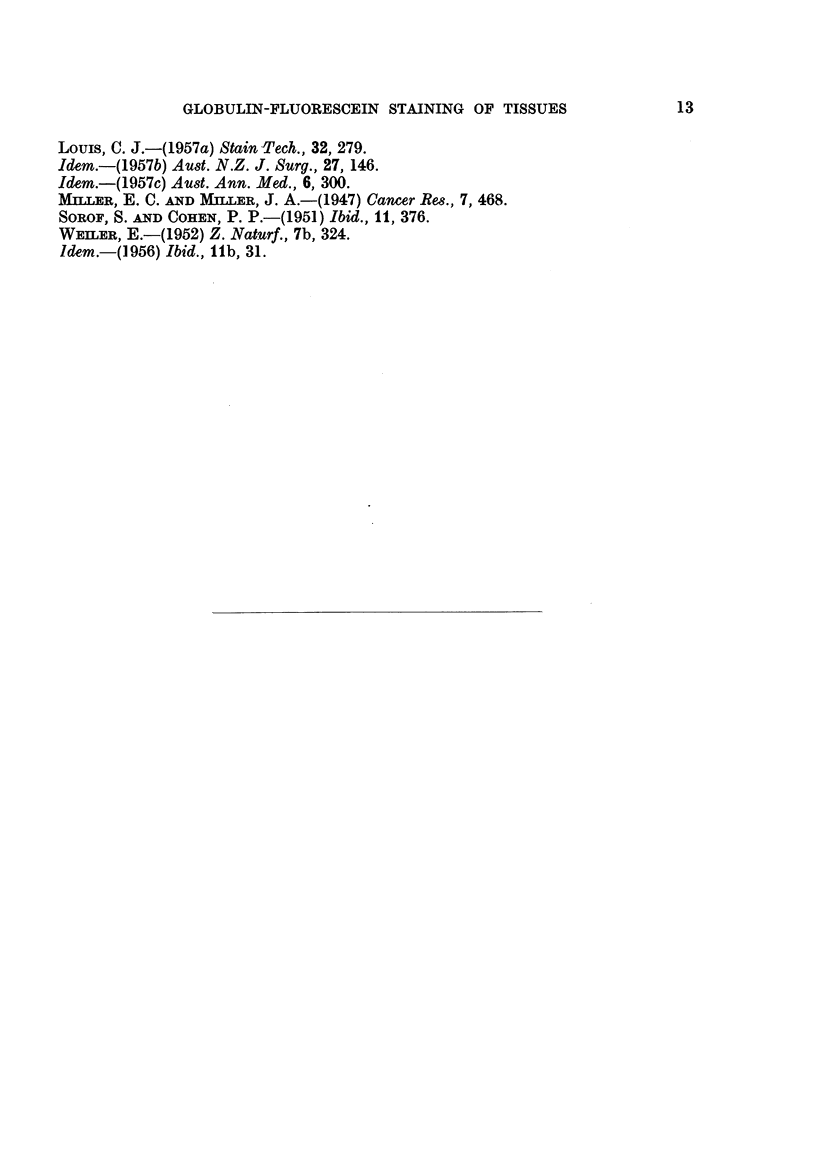# The Species non-Specificity of Globulins in the Globulin-Fluorescein Staining of Tissues

**DOI:** 10.1038/bjc.1958.2

**Published:** 1958-03

**Authors:** E. S. J. King, P. E. Hughes, C. J. Louis

## Abstract

**Images:**


### THE SPECIES NON-SPECIFICITY OF GLOBULINS IN THE GLOBULIN-FLUORESCEIN STAINING OF TISSUES

E. S. J. KING, P. E. HUGHES AND C. J. LOUIS

*From the Department of Pathology, University of Melbourne, Melbourne, Australia*

Received for publication November 27, 1957

MANY observers have suggested that the malignant cell differs from its normal counterpart in the loss or modification of a growth-controlling component but it was not until the work of Miller and Miller (1947) that firm experimental support for such an hypothesis was advanced. This work indicated that, at least as far as rat liver is concerned, the cellular constituent involved is protein in nature.

The results of Weiler's observations (1952, 1956) provided independent serological and histological support for the concept of the loss of a cellular constituent during aminoazo dye carcinogenesis in the rat liver. Weiler injected particulate antigens prepared from rat liver into rabbits and obtained antisera which, after repeated absorption with rat kidney particulates, still reacted in complement fixation test with the homologous rat liver but failed to react with rat kidney particulates. He found that a serum prepared in this way, which he regarded as a liver " organ-specific antiserum ", did not react with particulate diagnostic " antigens " prepared from rat hepatomata and concluded that the tumour cells had lost their " organ-specific antigen ". Further support of these results was obtained by application of the fluorescence antibody technique of Coons and Kaplan (1950). Weiler found that fluorescein-globulin conjugates prepared from " organ-specific anti-rat liver " rabbit globulin stained normal rat liver sections but did not stain sections of rat hepatoma.

Hughes, Louis, Dineen and Spector (1957), using Weiler's methods, were unable to obtain an organ-specific antiserum. At the same time the differential staining of normal rat liver and rat hepatoma sections by the use of a fluorescein-conjugated rabbit globulin fraction was completely confirmed (Hughes, 1957). At first serum obtained from rabbits which had previously been injected with homogenates of rat tissues was used. However, it was found that serum from rabbits which had not been so injected was equally effective as a stain. These control sera did not fix complement in the presence of rat liver antigens.

The method was applied to naturally-occurring tumours both in man and in animals. At first the sera from rabbits which had been injected with the appropriate tissues were used, but these were soon replaced by sera from uninjected animals. The technique was applied to various tumours and particularly to a series of carcinoma of the colon (Louis, 1957*b*) and it was also applied to a large series of cases of leukaemia (Louis, 1957*c*).

In view of these results it was clear that the staining could not be attributed to an antibody-antigen reaction and an alternative explanation was sought. In mixtures of proteins, anion-cation salt-like complexes are readily formed within the pH range between the isoelectric points of the components. The formation of these complexes between " stain globulin " and tissue proteins could account for these staining phenomena.

Since the $\gamma$-globulins as a whole represent a relatively homogeneous class of proteins the staining reactions of dye-globulin conjugates of various animals have been studied to determine if this staining property is a peculiarity of rabbit globulin or if it is a common property of globulin conjugates prepared from different genera, orders or even classes.

The results of an investigation using globulin conjugates of diverse origin are presented here. Most globulin fractions used were $\gamma$-globulin fractions prepared by cold ethanolic fractionation ; some other crude globulin fractions were prepared by ammonium sulphate fractionation. Globulin conjugates were prepared with both fluorescein and rhodamine.

### MATERIALS AND METHODS

### (1) *Preparation of globulin fractions*

Fowl, mouse and some rabbit globulin fractions were prepared by precipitation with 50 per cent saturated ammonium sulphate at 1° C. and subsequent dialysis against phosphate buffered saline pH 7·2.

Human $\gamma$-globulin, prepared by method 6 of Cohn, Strong, Hughes, Mulford, Ashworth, Melin and Taylor (1946), was obtained from the Commonwealth Serum Laboratories, Melbourne. $\Gamma$-globulin fractions of pig, dog, cat, horse, sheep, monkey and bovine sera were prepared using method 10 of Cohn, Gurd, Surgenor, Barnes, Brown, Derouaux, Gillespie, Kahnt, Lever, Liu, Mittelman, Mouton, Schmid and Uroma (1950). Corresponding fractions of rabbit, rat and guinea-pig sera were prepared by the modification of method 10 introduced by Goldstein and Anderson (1957).

### (2) *Preparation of fluorescent amines*

Fluorescein amine isomers were prepared by the method of Coons and Kaplan (1950).

Nitrorhodamine was prepared by heating equal parts by weight of *m*-NN diethylaminophenol and 4-nitrophthalic acid at 170–174° C. until the mass was dry (3–4 hours). After cooling, the melt was chipped from the beaker, ground to a powder and allowed to stand overnight in 1 N ammonium hydroxide. After filtering and drying the resultant crude nitrorhodamine was suspended in absolute ethanol and reduced to the amine with hydrogen at room temperature and atmospheric pressure in the presence of Raney nickel catalyst.

The Raney nickel was removed by centrifugation and washed with a small volume of ethanol. Addition of 4 volumes of water to the ethanolic solution caused precipitation of the amine. The precipitate was found to be too fine to filter by ordinary methods and slow to separate on centrifugation. Therefore it was extracted with a small volume of ethylacetate and crystallized from the solvent.

Fluorescein amine and rhodamine amine were converted to the corresponding isocyanates by treatment with phosgene under anhydrous conditions and conjugated with the appropriate globulin fractions by the method of Coons and Kaplan (1950). Following conjugation dioxane, acetone and excess free fluorescein or rhodamine derivatives were removed by ethyl acetate extraction (Dineen and Ada, 1957) prior to use.

Sections of tissue, where practicable, were taken from the margins of various tumours, prepared by a method previously described (Louis, 1957*a*) and were treated with the appropriate stain for 10 minutes at room temperature. The sections were washed in three changes of phosphate buffered saline for 10 minutes prior to examination. For microscopy a Leitz fluorescence microscope fitted with two BG 4 mm. filters between light source and condenser and a Wratten G 15 gelatin filter in the ocular was used.

### RESULTS

Sections of each of a large series of tumours, both innocent and malignant, were individually treated with the above enumerated stains. In all cases identical staining affinities were found for a particular tissue regardless of which animal provided the globulin used or whether a fluorescein or rhodamine conjugate was employed to enable visualization of the globulin; the results are presented in tabular form.

The results of the series of human tumours investigated are shown in Table I and those of animals in Table II.

TABLE I

Results obtained by staining human tissues with conjugated globulins. Both fluorescein and rhodamine were used but, since there was no appreciable difference between the two regarding intensity and type of staining, these were not given separately. In all cases the results were comparable, that is non-neoplastic tissue stained and fluoresced well but malignant neoplastic tissue did not; nevertheless the numbers and types of cases examined are set out in detail. The globulins of all the sera (other than rabbit) were examined in groups so that the figures (number of cases) apply to all the genera cited. (+) = positive staining, (−) = absence of staining.

| Human material examined | | Results obtained with different globulins | |
|---|---|---|---|
| Tissue | Condition | Normal rabbit | Rat, guinea-pig, pig, mouse, horse, cattle, sheep, man, monkey, dog, cat, fowl |
| Skin | Normal epithelium | (+)28 | (+)7 |
| | Molluscum pseudo-carcinomatosum | (+) 6 | (+)3 |
| | Squamous-cell carcinoma | (−) 8 | (−)4 |
| Breast | Hyperplasia | (+)54 | (+)6 |
| | Fibroadenoma | (+) 7 | (+)3 |
| | Carcinoma | (−)26 | (−)6 |
| Stomach | Normal mucosa | (+) 8 | (+)5 |
| | Carcinoma | (−) 5 | (−)3 |
| Colon | Normal mucosa | (+)32 | (+)3 |
| | Polyp | (+) 2 | (+)2 |
| | Carcinoma | (−)28 | (−)3 |
| Lung | Bronchial mucosa | (+)12 | (+)3 |
| | Carcinoma | (−) 9 | (−)3 |
| Thyroid | Normal | (+)26 | (+)5 |
| | Colloid goitre | (+)18 | (+)5 |
| | Foetal adenoma | (+) 8 | (+)3 |
| Lymph node | Normal lymphoid tissue | (+)10 | (+)3 |
| | Secondary melanoma | (−) 1 | (−)1 |
| | Secondary carcinoma, breast | (−) 5 | (−)2 |

Representative examples of these cases are illustrated in Fig. 1–9. These have been chosen, at random, from a large series of specimens which have been examined and photographed. It can be seen that the innocent tissue in these illustrations fluoresces whilst the neoplastic tissue does not fluoresce. In the so-called innocent tumours both the normal and pathological tissues fluoresce. Preneoplastic rat liver resulting from the feeding of 4-dimethylaminoazobenzene displayed islands of morphologically normal parenchymal cells with decreased

---

### EXPLANATION OF PLATES

FIG. 1.—Photomicrograph (with ultra-violet light) of a frozen section of margin of a *rat* hepatoma induced by feeding 3′-methyl-4-dimethylaminoazobenzene showing fluorescence of cytoplasm of normal cells and absence of fluorescence of cytoplasm of neoplastic cells. Stain—fluorescein-fowl globulin complex.

FIG. 2.—Photomicrograph showing the same area as in Fig. 1 stained with haematoxylin and eosin ; the structure of the non-fluorescing area is demonstrated.

FIG. 3.—Photomicrograph (with ultra-violet light) of a frozen section of the margin of a *rat* cholangioma induced by feeding 3′-methyl-4-dimethylaminoazobenzene and showing fluorescence of cytoplasm of normal cells and absence of fluorescence of neoplastic cells. Stain—fluorescein-human globulin complex.

FIG. 4.—Photomicrograph showing the same area as in Fig. 3 stained with haematoxylin and eosin ; the glandular and neoplastic character of the non-fluorescing area is demonstrated.

FIG. 5.—Photomicrograph of a frozen section of *rat* liver after feeding 4-dimethylaminoazobenzene for 6 weeks showing an island of parenchymal cells which have lost their affinity for the stain. Stain—rhodamine-rabbit globulin complex.

FIG. 6.—Photomicrograph of the same section as in Fig. 5 stained with haematoxylin and eosin showing the morphology of the non-staining cells.

FIG. 7.—Photomicrograph of a frozen section of the margin of a spheroidal cell carcinoma of the stomach (*man*) showing fluorescence of the cytoplasm of normal cells and absence of fluorescence of the neoplastic cells. Stain—fluorescein-dog globulin complex.

FIG. 8.—Photomicrograph of the same area as in Fig. 7 stained with haematoxylin and eosin ; the structure of the non-staining area is that of a typical spheroidal cell carcinoma.

FIG. 9.—Photomicrograph of a frozen section prepared from a mixed salivary tumour (*man*) showing fluorescence of epithelial cells in cellular areas as well as those in the mucoid areas. Stain—fluorescein-monkey (rhesus) globulin complex.

FIG. 10.—Photomicrograph of the same area as in Fig. 9 stained with haematoxylin and eosin to show the morphological characters of the fluorescing cells. These appearances indicate this growth (at this stage) corresponds with the non-neoplastic rather than the neoplastic conditions.

FIG. 11.—Photomicrograph of a frozen section prepared from a *human* lymph node containing secondary melanomatous deposits showing lack of fluorescence in the secondary deposits. Stain—fluorescein-mouse globulin complex.

FIG. 12.—Photomicrograph of same area as in Fig. 11 stained with haematoxylin and eosin showing that both melanotic and amelanotic tumour areas do not fluoresce. The fluorescing area in Fig. 11 is a collection of reticulo-endothelial cells.

FIG. 13.—Photomicrograph of a frozen section of a transplanted carcinoma of the breast in a *mouse* showing fluorescence of adjacent skin but absence of fluorescence in the subjacent area. Stain—fluorescein bovine globulin complex.

FIG. 14.—Photomicrograph of the same area as in Fig. 13 stained with haematoxylin and eosin showing the structure of the non-fluorescing area to be that of a typical epithelial (carcinomatous) tumour.

FIG. 15.—Photomicrograph of a blood film taken from a *mouse* suffering from chronic lymphatic leukaemia showing fluorescence of leucocytes and absence of fluorescence of erythrocytes. Stain—fluorescein-sheep globulin complex.

FIG. 16.—Photomicrograph of same area as in Fig. 15 stained by Leishman's method, showing that all the leucocytes fluoresce.

FIG. 17.—Photomicrograph of frozen section of a fibroadenoma of the *human* breast showing fluorescence of the epithelial cells lining the ducts. Stain—fluorescein-horse globulin complex.

FIG. 18.—Photomicrograph of same area as in Fig. 17 stained with haematoxylin and eosin.

All magnifications × 120.

1

2

3

4

5

6

King, Hughes, and Louis.

7

8

9

10

11

12

13

14

15

16

17

18

### Table II

Results obtained by staining various tissues (normal and tumour) with different globulin complexes. Both fluorescein and rhodamine were used (see Table I). Although exceptions to the staining characters described were not found, the results are set out in detail to indicate the actual extent of the investigation to date. Figures give number of cases examined; + = positive staining, — = absence of staining.

| | | Results obtained with globulins from different species (No. of cases examined) | | | | | | | | | | | | |
|---|---|---|---|---|---|---|---|---|---|---|---|---|---|---|
| Animal | Conditon | Rabbit | G.-pig | Rat | Mouse | Pig | Horse | Cattle | Sheep | Man | Mon-key | Dog | Cat | Fowl |
| Mouse | Skin (normal) | +10 | +3 | +3 | +5 | +2 | +2 | +2 | +2 | +3 | +3 | +2 | +2 | +3 |
| | Liver (normal) | +6 | +1 | +1 | +1 | +1 | +2 | +2 | +1 | +1 | +1 | +1 | +1 | +2 |
| | W.B.C. (normal) | +56 | +5 | +5 | +6 | +3 | +2 | +3 | +2 | +4 | +2 | +2 | +2 | +7 |
| | R.B.C. (normal) | −56 | −5 | −5 | −6 | −2 | −2 | −3 | −2 | −4 | −2 | −2 | −2 | −7 |
| | Spont. ca. breast | −1 | −1 | −1 | −1 | −7 | −1 | −1 | −1 | −1 | −1 | −1 | −1 | −1 |
| | Transpl. ca. breast | −7 | −2 | −2 | −4 | −1 | −1 | −2 | −1 | −2 | −1 | −1 | −1 | −2 |
| | W.B.C. (ac. leukaemia) | −28 | −3 | −3 | −4 | −2 | −1 | −2 | −1 | −2 | −1 | −1 | −1 | −4 |
| Rat | Liver (normal) | +48 | +1 | +3 | +1 | +1 | +1 | +2 | +1 | +13 | +1 | +1 | +1 | +3 |
| | Hepatoma | −6 | −2 | −2 | −2 | −2 | −2 | −2 | −2 | −6 | −2 | −2 | −2 | −2 |
| | Cholangioma | −4 | −2 | −2 | −2 | −2 | −2 | −2 | −2 | −4 | −2 | −2 | −2 | −2 |
| | Ca. breast | −3 | −1 | −3 | −1 | −1 | −1 | −2 | −1 | −1 | −1 | −1 | −1 | −2 |
| Fowl | Liver (normal) | +2 | +1 | +1 | +2 | +1 | +2 | +2 | +1 | +2 | +1 | +1 | +1 | +2 |
| | W.B.C. (normal) | +3 | +1 | +1 | +2 | +2 | +1 | +2 | +1 | +2 | +1 | +1 | +1 | +2 |
| | R.B.C. (nuclei) | +3 | +1 | +1 | +2 | +2 | +1 | +2 | +1 | +2 | +1 | +1 | +1 | +2 |
| Cattle | Liver (normal) | +1 | +1 | +1 | +1 | +1 | +1 | +1 | +1 | +1 | +1 | +1 | +1 | +1 |
| | Second ca. liver | −1 | −1 | −1 | −1 | −1 | −1 | −1 | −1 | −1 | −1 | −1 | −1 | −1 |
| | Ca. adrenal | −1 | −1 | −1 | −1 | −1 | −1 | −1 | −1 | −1 | −1 | −1 | −1 | −1 |

affinities for both fluorescein and rhodamine conjugates of the various globulins (Fig. 5, 6).

No significant difference in intensity of staining was found with the different globulins. Modifications of technique were not employed, that is to say, the same staining times under strictly comparable conditions were used in all cases. The degree of fluorescence both as observed by the eye and as recorded on the photographic plate was of the same, or at least comparable, degree in all cases.

The intensity of fluorescence observed in these studies is not directly comparable with that found when dealing with specific fluorescent antibody-antigen reactions. It is a more general type of fluorescence classed as non-specific by Coons and Kaplan (1950) so that from these results it may be stated that normal as opposed to malignant tissue has an affinity for the component of the stain giving, immunologically speaking, non-specific fluorescence. Repeated absorption of these sera with acetone-dried mouse liver particles removes this type of fluorescence staining completely. As with any other form of staining with tissues, certain conditions have to be met to give satisfactory cytological differentiation. In the present case unconjugated fluorescein derivatives must be removed. This has been accomplished by ethyl acetate extraction of the sera since this method preserves the active component of the stain.

### DISCUSSION

In this investigation we have found that the staining characteristics of conjugates prepared from several globulins have been identical, regardless of the species of origin of the globulin. The result is thus independent either of any species variations in the globulins tested or in the means of serum fractionation employed in obtaining the required fractions.

It is known that the globulins of different orders differ considerably; for example, those of the larger ungulates, such as the horse and cattle, have a much higher molecular weight than the globulins of some of the other animals. Thus it might have been expected that these would act differently but, in fact, both from the point of view of their formation of fluorescein and rhodamine conjugates and also their reaction with various types of tissues, they were essentially similar to those of other animals. This, therefore, indicates that the characteristics of the stain complex are due to features other than the molecular weight of the globulin or any special peculiarity of its intramolecular structure.

In the early stages of the investigation, rabbits were injected with homogenates of tissues of various kinds from various animals. When the question was raised as to the significance of the antigen-antibody reaction, some of the presumable "specific antibody globulin" for one tissue was used for some other, for example, globulin from a rabbit which had been injected with colon mucosa as the antigen was used for breast tissue. It was found that it produced as distinctive differentiating staining as occurred when the globulin from an animal injected with the appropriate tissue was employed.

Various methods of fractionation of the proteins of the serum were employed but no significant difference was found in the staining of these various protein conjugates. This would suggest that the staining phenomenon described is not peculiar to one particular type of protein and that the effect might be produced by a much wider range of proteins than was previously expected. This will be a matter for future study but, for the present, it is apparent that not only the $\gamma$-globulins but the immediately related globulins also act in a similar manner. This further supports the suggestion that the phenomenon is not a serological antigen-antibody reaction but rather some physico-chemical interaction due to the physical properties (possibly those of the electric charge) of the serum proteins.

Sorof and Cohen (1951) have shown, in a variety of tumours, that there is a significant reduction in the proportion of the more basic cytoplasmic proteins. These proteins, because of their extreme basicity, could be expected to form complexes with globulins selectively over a wide range of pH. Such differential staining, as has been observed, is therefore not unexpected in view of these electrophoretic findings.

The results obtained, moreover, are in entire agreement with such a view. The $\gamma$-globulins form a relatively homogeneous class of protein and if anion-cation complex formation is involved they could be expected to behave similarly because of similarities in their isoelectric points and electric charges. Of special significance are the observations that the homologous globulin is equally efficacious in obtaining the staining as any heterologous globulin. Thus human tissues stain equally well with human $\gamma$-globulin conjugates as with those prepared from any other animal.

Since similar differential staining could be achieved with either fluorescein or rhodamine conjugates the individuality of the dye moiety could not be implicated. Thus it can be seen that the $\gamma$-globulins as a class of proteins are all equally suitable for demonstrating this staining reaction.

There are a few points of special technical importance. When tissues are exposed to ultra-violet light some of them will demonstrate a fluorescence which is peculiar to them themselves. This is quite different and distinct from that of the stains which have been used but, nevertheless, requires to be specially differentiated. This autofluorescence occurs particularly in elastic tissue and in keratin and it has, here, a characteristic yellow and bluish-white colour respectively. This is quite distinct from the green colour of the fluorescein or the orange of the rhodamine but, in any case, can be readily differentiated by first examining the section under the ultra-violet light without any staining.

We have found that not all normal cells stain by this method. The cells which do stain are normal epithelial cells and some phagocytic cells. Neoplastic cells, whatever their origin, do not stain. This is clearly a property of the neoplastic cell and is not a function of the amount of the cytoplasm in the cell. Normal connective tissue cells, possibly by view of decreased cytoplasm, do not stain. It would seem that the intercellular substance does not contain the basic protein complex which is the characteristic of the cytoplasm of the epithelial cells.

This investigation is still in its early stages and much information concerning the types and characteristics of proteins of various kinds of cells and of the intercellular substance of the various connective tissues is required before far-reaching conclusions can be drawn. However, for the time being, it seems clear that most epithelial cells contain, amongst the variety of the cytoplasmic proteins, a basic protein which, from the point of view of the present study, is the focal point of this staining reaction. This protein appears to be present also in some of the connective tissue cells, particularly some of the wandering cells and endothelial cells which possess a considerable amount of cytoplasm in their structure.

While many characteristics of tumour cells have been described, none has been found to be peculiar *per se* to the neoplastic state. The pathologist has always had to resort to the general architecture of the tissue for a true assessment of the condition. Here, however, we have a clearly defined feature which distinguishes sharply the tumour cell from its normal counterpart. At first it seemed as if this was a characteristic peculiar to certain chemical carcinogen-induced neoplasms and that the phenomenon was a complex biological antigen-antibody reaction. The multiplicity of the globulins which show the characteristic staining reaction (and particularly the applicability of the homologous globulins in this regard) demonstrates the relatively simple physico-chemical interrelation between proteins of different electrical charges which underly this phenomenon.

The ability of this protein of the normal cells to form complexes with fluorescein-globulin conjugates selectively (and thus to be stained selectively) and conversely the inability of malignant cells, which lack the protein, to demonstrate any such staining appears to be a feature by which individual neoplastic cells may be recognized. Not only is this a matter of prime importance, as a method of investigation, in the recognition of malignant tissue in cases where its normal recognition is difficult but it emphasizes an important distinction between the malignant and the normal cell and this has already proved to be a crucial difference which has far-reaching implications in relation to several aspects of basic cancer research.

### SUMMARY

Following injection of rabbits with homogenates of rat tissue, a rabbit $\gamma$-globulin was shown to combine with normal rat tissue cells but not with tumour cells of the same organ. This was demonstrated visually by the use of a globulin-fluorescein complex which fluoresced in ultra-violet light.

Although at first regarded as an antigen-antibody reaction it was shown, by the demonstration of the same characteristics when normal rabbit globulin was used instead of that from an injected rabbit, to be a protein interaction of no serological significance.

This feature was examined first in the case of tumours produced in animals following the administration of chemical carcinogens but was applied subsequently to naturally occurring tumours of both animals and man.

The method has now been employed also using globulins from normal mouse, rat, guinea-pig, horse, cow, sheep, pig, dog, cat, monkey, man and fowl. In all cases the results obtained were so similar as to be indistinguishable in degree and kind from those observed with the rabbit sera.

The method of production of the globulin was different in different cases and in the rabbit more than one method was employed. No difference was observed in the results.

The stains were prepared by conjugating the globulins with both fluorescein and rhodamine without any significant difference (other than colour) being seen in the staining effects.

It appears, therefore, that the results observed (within the range of animals examined) are due to physico-chemical characteristics of globulin irrespective of the species from which it is obtained. The phenomenon of staining of normal tissue and non-staining of malignant tissue, therefore, by the use of globulin-fluorescein conjugates, is a physico-chemical interaction depending on peculiarities other than those of a serological character.

We are indebted to the Commonwealth Serum Laboratories for several of the serum fractions ; technical assistance with the conjugation of globulins provided by Staff Members of the Defence Research Laboratories, Melbourne, is gratefully acknowledged ; and thanks are tendered to Mr. W. R. Mackay, Assistant Pharmacist, Royal Melbourne Hospital, for cheerful aid in various stages of the work. This work was supported by the Anti-Cancer Council of Victoria.

### REFERENCES

COHN, E. J., STRONG, L. E., HUGHES, W. L., Jr., MULFORD, D. J., ASHWORTH, J. N., MELIN, M. AND TAYLOR, H. L.—(1946) *J. Amer. chem. Soc.*, **68**, 459.

*Idem*, GURD, F. R. N., SURGENOR, D. M., BARNES, B. A., BROWN, R. K., DEROUAUX, G., GILLESPIE, J. M., KAHNT, F. W., LEVER, W. F., LIU, C. H., MITTELMAN, D., MOUTON, R. F., SCHMID, K. AND UROMA, E.—(1950) *Ibid.*, **72**, 465.

COONS, A. H. AND KAPLAN, M. H.—(1950) *J. exp. Med.*, **91**, 1.

DINEEN, J. K. AND ADA, G. L.—(1957) *Nature, Lond.*, **180**, 1284.

GOLDSTEIN, J. AND ANDERSON, J. W.—(1957) *J. biol. Chem.*, **224**, 775.

HUGHES, P. E., LOUIS, C. J., DINEEN, J. K. AND SPECTOR, W. G.—(1957) *Nature, Lond.*, **180**, 289.

*Idem.*—(1957) *Aust. N.Z. J. Surg.*, **26**, 302.

LOUIS, C. J.—(1957*a*) *Stain Tech.*, **32**, 279.
*Idem.*—(1957*b*) *Aust. N.Z. J. Surg.*, **27**, 146.
*Idem.*—(1957*c*) *Aust. Ann. Med.*, **6**, 300.
MILLER, E. C. AND MILLER, J. A.—(1947) *Cancer Res.*, **7**, 468.
SOROF, S. AND COHEN, P. P.—(1951) *Ibid.*, **11**, 376.
WEILER, E.—(1952) *Z. Naturf.*, **7b**, 324.
*Idem.*—(1956) *Ibid.*, **11b**, 31.